# The Eye and Its Treatment

**Published:** 1873-11

**Authors:** J. W. Wright

**Affiliations:** Coshocton, Ohio


### SELECTIONS.

---

### THE EYE AND ITS TREATMENT.

BY J. W. WRIGHT, COSHOCTON, OHIO.

So little study and attention is given to the diseases of the eye and their treatment by the general practitioner, that a few words on the management of some of the most common affections may prove beneficial to the physician. Having for the past eight years made the diseases of the eye a study and practice, I can readily refer to several cases of blindness caused either by a neglect on the part of the patient, or the improper treatment by the physician in charge of the case.

The eye is affected with so many diseases, and there is so much study necessary to make one proficient in the special treatment of its disorders, that it is not to be wondered at that the general practitioner is not well prepared for all cases; but there are some certain affections so frequent, and so dangerously destructive to sight, that it is surprising that they are not better studied and managed.

*Inflammation of the eyes—or sore eyes,* as we often hear the expression—is no definition for any particular disease of that organ, for almost any disease of the eye will cause an *inflammation* of it and make it *sore.* Yet we find in almost every neighborhood, one or two persons who have a positive cure for "sore eyes," and I am sorry to say, we find physicians occasionally, who prescribe some favorite collyrium in all cases of the disease.

Of the various methods of treatment the local is most frequently used, consisting of collyria, more or less astringent, and almost always containing acetate of lead—commonly called *sugar of lead*—and other ingredients equally obnoxious. Acetate of lead is very dangerous unless used by one who understands what he is doing. It is not a remedy to be used at random. When ulceration of the cornea exists, the lead is liable to be deposited in the texture of the cornea, forming an opacity. The use of strong solutions of the nitrate of silver is subject to the same objections. I have known the incautious use of it to cause partial destruction of the folds of the conjunctiva, and adhesions between the lid and the globe. I do not wish it understood that these remedies should never be used, but I would guard the general practitioner against the incautious use of them or any other strong, irritating washes; for I am very positive that many an eye has been destroyed by just such harsh treatment.

When you are called upon for treatment in any eye trouble, you should make a strict examination of the member and its appendages. First, get a view of the globe, and ascertain if possible, if there is any foreign substance in the eye. You will not be able to say to the contrary until you evert the lids, when very often you will find the source of trouble attached to the lid itself. If so, remove it at once. Give some mild collyrium, as morphia grs. v. to aqua pura ℥ i; apply four or five drops every two or three hours, and advise the patient to bathe the eye frequently with cold water; and on retiring to bed to have a basin of cold water within his reach, and whenever the eye feels feverish to apply cloths immersed in the water to it, changing them frequently. Generally this is all the treatment necessary. But there are other cases where a foreign substance enters the eye, or the eye is struck forcibly by some substance in such a manner as to cause a slight abrasion of the cornea, that the trouble does not end so easily or so soon. I have known slight abrasions of the cornea produce little or no trouble; but there are times, owing to the condition of the patient, when even a very slight abrasion causes a vast amount of pain, and very frequently the loss of an eye. If we find an ulcer beginning from such a cause then we must give the eye close attention.

While speaking of ulcers of the cornea, do not understand that all ulcers are caused by foreign substances. They have various causes. There is an ulcer of the cornea, common in children, of a strumous diathesis, from two to twelve years of age. We also often see other children, more healthy, when subject to cold and damp weather, and especially during teething, attacked with ulcers of the cornea. In these cases we rarely find both eyes affected similarly at the same time, but the symptoms often alternate—first on one, then on the other. Ulceration of the cornea may also be the result of granular lids, purulent ophthalmia, and other diseases.

In the treatment of ulceration, constitutional remedies will be found of very great importance. Tonics consisting of the preparations of iron, of which the solution of iodide of iron with quinia, has been highly recommended. I am not able to say whether the iodide is any better than the citrate of iron with quinia. I have always found the latter very good. Be that as it may, we must use good tonics, enforce nutritious diet and warm clothing. A cathartic might be given at the start, but the plan of giving man or child frequent purges and enforcing a weak diet, with a view to cleanse the blood, is pernicious, and is only giving the ulcer a better chance to destroy the cornea. We should, if possible, touch the ulcer with a pencil of the sulphate of copper, or *blue stone* as it is frequently called, but we should *merely touch* it, and that not oftener than once a day or every two days.

If from the extreme sensitiveness of the eye, and especially if the patient be a child, we often have trouble in making direct application to the ulcer. In such cases we can raise the upper lid a little and merely rub the under surface of it with the pencil, when the medicine will come in contact with the ulcer.

This application is of more benefit than any collyria that could be used, and especially one containing nitrate of silver or acetate of lead, in fact, just such cases forbid the use of such remedies. But I can recommend a collyrium that will be of great benefit, especially if there be photophobia. It is the solution of atropia, grs. ii to v, to aqua dura 1 oz. If there is much pain I add morphia sulph. grs. v to x, and we have an anodyne that in my opinion cannot be excelled. I am satisfied that a great many eyes have been entirely destroyed, just because the use of atropia has been neglected. You may be positive of one thing—you will have a very few, if any cases of staphylomia irridis after ulceration, if you have applied the atropia frequently.

A young man made application to me not long since with ulceration of the cornea. He had not injured his eye in any way, nor had disease of the organ that he knew of previously; however, his general health was poor, appetite bad, and bowels irregular. The ulcer had so far progressed that the iris had become attached to the cornea at the point of the ulcer, (*Synache Anterior*). The ulcer was large. There was considerable inflammation of the conjunctiva, great pain and lachrymation. I commenced treatment by giving him compound cathartic pills, enough to move his bowels quite freely; I then gave him the tonic of citrate of iron and quinine, touched the ulcer once a week for three weeks, (I did not see him oftener) and kept the solution of atropia constantly to his eye. Besides this, I used a cup to his temple, not drawing any blood, and had him apply it every day while at his home, until the pain was banished. At the end of the third week I told the young man that his eye was as well as it would ever be, the ulcer was entirely gone, but the iris was still attached to that point in the cornea. I told him that he might keep up the use of the atropia and perhaps by keeping the iris on a stretch he might break the attachment, however, I had no idea such would be the case. He continued the use according to my directions. In two weeks he called again and happily the atropia had its effects. The iris was released, and the pupil looked almost as natural as ever; the cornea was almost opaque, but in time it will become very nearly as clear as ever.—*Cin. Med. News.*

---

Dr. Abner Phelps died in Boston, Feb. 24th, aged 95 years. In 1826, when a member of the Legislature, he proposed the first rail road bill ever drafted in America.

